# An empirical study of how the Dutch healthcare regulator first formulates the concept of trust and then puts it into practice

**DOI:** 10.1186/s12913-019-4797-3

**Published:** 2019-12-10

**Authors:** Sandra Spronk, Annemiek Stoopendaal, Paul B. M. Robben

**Affiliations:** 1 Health and Youth Care Inspectorate, Stadsplateau 1, 3521 AZ Utrecht, The Netherlands; 2 Erasmus School of Health Policy & Management, Burgemeester Oudlaan 50/, 3062 PA Rotterdam, The Netherlands

**Keywords:** Trust, Regulation, Inspector, Inspectee, Behavior, Context

## Abstract

**Background:**

Responsive regulation assumes that the parties being regulated are trustworthy and motivated by social responsibility. This assumes that regulation based upon trust will improve the regulated organization more effectively than other regulation models. The purpose of our qualitative study was to unravel the most important elements of trust in the inspectee which can support the inspector’s work and to develop a model and a framework of trust that can be used by the inspectors to legitimize their trust in the inspectee.

**Methods:**

We conducted an empirical study on trust regarding the regulation of care services to reveal how trust in the inspectee is conceptualized and assessed. Based on literature and empirical research, we synthesized the concept of trust into six elements, five regarding behavior, and a sixth looking at information about its context. We developed a practical framework for the concept to reduce the conceptual ambiguity, strengthen regulatory assessment, and support appropriate tailoring of the regulatory response.

**Results:**

Six elements with respect to trust emerged from the data: showing integrity; transparency; ability to learn; accepting feedback; showing actual change in behavior; context information. These five behavioral elements, plus the context information were merged into a Framework of Trust and designed into an interactive PDF document.

**Conclusions:**

This study has sought to address a gap in the empirical knowledge regarding the assessment of trust in the inspectee. The results aim to inform and clarify the regulatory conceptualization and understanding of trust in the inspectee. Other inspectorates may learn from these results for their own practice and explore whether operational deployment of our Framework of Trust effects their assessment and enforcement strategies.

## Background

Responsive regulation has become the foremost approach since its introduction by Ayres and Braithwaite in 1992 [1, 2]. This regulation [1] emphasizes the combination of both ‘deterrence’ [3] and ‘compliance’ [4] models. It is a flexible model which allows regulators to choose their approach depending on performance or levels of risk [5]. Regulatory intervention escalates, or de-escalates, through a hierarchy as performance or the level of risk changes. In addition, responsive regulation stresses the importance of trust. This form of regulation assumes that the regulated parties are trustworthy and motivated by social responsibility, and it will, therefore, improve the organization more effectively [1, 2].

Various types of the responsive model have followed, such as ‘smart’ regulation [6] or risk-based regulation [7]. Proponents of smart regulation argue that responsive regulatory strategies can be enhanced through ‘self-regulation’, whereby organizations review their own performance through the use of third parties, such as accreditation bodies. Proponents of smart regulation suggest that it encourages self-reflection about performance, and thus ongoing improvement. Proponents of risk-based regulation argue that regulators, in principle, should focus their efforts on the most serious risks that they face in achieving their objectives. This risk-based regulation runs parallel to Malcolm Sparrow’s exhortation that regulators should `pick important problems and fix them’ [8]. This form of regulation gives priority to matters that are serious and important [9].

The Dutch Health and Youth Care Inspectorate (hereafter, the Inspectorate) is the official regulatory body charged with safeguarding the quality of care services, prevention activities and medical products. The Inspectorate will take action against any care provider or manufacturer who fails to comply with current legislation. Its main approach is risk-based regulation, whereby it focuses on sectors and activities in which the risks are greatest, as identified by a system of risk analysis. General information about the Inspectorate and its work can be found in Additional file 1. In addition, the Inspectorate embraces responsive regulation, accepting the idea that, in the first instance, it is a dynamic regulatory strategy of dialogue and trust, and only followed by a more punitive regulation when trust is abused. This idea matches the growing emphasis by the Inspectorate on the organizational learning and safety culture of the inspectee [10]. Trust is important to safety culture because it affects matters related to safety such as communication, collaboration, sharing information, and the reporting of incidents or near misses [11]. Furthermore, trust is important to a learning which is characterized by the importance of continuous reflection in order to assess performance [12].

Trust, however, remains ambivalent. Ultimately, it is dependent on the inspector’s leap of faith [13]. Although the literature covers a wide variety of topics on trust, the literature regarding elements of trust, in particular for regulation, is limited. The inspectors, therefore, have insufficient tools and guidance to use the concept of trust explicitly and in a consistent manner.

Our research question is: ‘How, in their daily practice, do inspectors interpret and use the concept of trust in the inspectee?’

We defined the following objectives:

- To understand and define trust from the inspector in the inspectee;
- To unravel the most important elements of trust in the inspectee which can support the inspector’s work;
- To develop a model and a framework of trust that can be used by the inspectors to legitimize their trust in the inspectee;
- To enable the Inspectorate to communicate on the issue of trust between inspectors and inspectees in a well-structured manner.

### Theoretical framework

We used, as a starting point, a recent semi-systematic literature review of trust in regulatory regimes [14]. This review allowed us to find other relevant papers on our subject. Our search was designed to provide insight into the elements of trust regarding regulation. Therefore, this section points out the definitions of trust used in these studies, which in turn enabled us to unravel the most important elements of trust that emerged.

There are several definitions of trust within the literature which have been derived from a range of theoretical perspectives. These definitions have different elements which are essential or characteristic parts of each definition (Table 1). However, many of the elements of trust in these definitions are interrelated and the boundaries are unclear [14].

**Table 1:** Definitions, descriptions, and elements from trust

Author (reference)	Definition or description	Elements
Rousseau [15]	Trust is a psychological state comprising of the intention to accept vulnerability based upon the positive expectations of the intentions or behavior of another.	Intentions Vulnerability Expectations Behavior
Alston [16]	Trust is a social expectation that has to do with people’s perception of the integrity, honesty, openness, caring, and competence of an individual or system that is verified by experiences.	Expectation Perception Integrity Honesty Openness Caring Competence Experiences
Adler [17]	Trust is the subjective probability with which an actor assesses that another actor or group of actors will perform a particular action, both before she or he can monitor such action (or independently of his or her capacity ever to be able to monitor is) and in a context in which it affects his or her own action.	Subjectivity Probability Capacity Context
Goudge [18]	Trust is a judgment in a situation of risk that the trustee will act in the best interest of the truster, or at least in ways that will not be harmful to the truster.	Situation of risk harmful
Gilson [19]	Trust is a relation notion that lies between people, people and organizations, and people and events.	relation
Schee van der [20]	Trust is being confident that you will be adequately treated when you are in need of healthcare.	confident Adequately treated
Dietz [21]	Trust is an assessment (however thorough) of the other party’s trustworthiness which informs a preparedness to be vulnerable that, in genuine cases of trust, leads to a risk-taking act.	Vulnerable Risk-taking act
Möllering [22]	Trust is a reflexive process of building on reason, routine and reflexivity, suspending irreducible social vulnerability and uncertainty *as if* they were favourably resolved, and maintaining a state of favourable expectation towards the actions and intentions of more or less specific others.	Reason Routine Reflexivity Vulnerability Uncertainty Expectation Actions Intentions
Nooteboom [23]	Trust is the expectation that people don’t let us down based on their intentions, competencies and the circumstances.^a^	Expectation Intentions Competencies Circumstances
Meurs [24]	Trust is based on past performance, competencies and intentions.^a^	Performance Competencies Intensions
Six [25]	There is trust if you are dependent of someone, for something that is important, that you can-not control completely, and can-not predict with certainty^a^.	Dependency Control Prediction Certainty

^a^translated from Dutch

A widely accepted and most frequently cited definition comes from Rousseau who wrote: ‘*Trust is a psychological state comprising of the intention to accept vulnerability based upon the positive expectations of the intentions or behavior of another’* [15]. One part of the definition is ‘the intention to accept vulnerability’, which means that at least one party in the relationship is vulnerable. Apparently, trust means making ourselves vulnerable. The other part of the definition refers to ‘positive expectations’, which means that it includes the expectation that the other party will perform a particular action. According to Dietz there is always an assessment of the trustee which informs the preparedness of the trustor to be vulnerable, which, in turn leads to a ´risk-taking act´ [21]. In line with Dietz, Goudge used the word ‘risk’ in the sense that trust is a judgement in a ‘situation of risk’ [18].

Most definitions converge on the idea that trust involves a trustor and trustee who are somehow interdependent and form a relationship [19, 25]. An actor trusts another actor with respect to their future behavior [26, 27]. Moreover, trust implies there is uncertainty about the trustee’s future behavior [15]. Simmel argues that it involves a ‘leap of faith’, which means believing in something whose existence or outcome cannot be proven or known [13]. Möllering’s idea is that trust is a prudent choice based on an assessment of the trustee’s trustworthiness, defined as benevolence, competence and/or integrity [22].

Trust often exists on an interpersonal level, but also on an organizational level, independent of specific individuals [17]. How these two levels, the interpersonal and organizational, connect and interact remains an interesting issue for regulation. Organizational trust can be defined as ‘organizational willingness, based upon its culture and communication behaviors in relationships’ [28]. This is the belief that another individual or group is open and honest [16]. Trust in people is called ‘behavioral trust’ [23]. Apparently, to look at trust in people is to look at the behaviors of people. Bidault and Jarillo have added the word ‘actual’ to the behavior of people as an element of trust [29]. Positive intentions toward the other is promising, but the actual behavior, that is whether the trustee fulfills their positive intentions, is paramount.

Furthermore, one has to distinguish between trust in how competent one is, and trust in what one’s intentions are [23]. These elements are part of trust under certain circumstances. In other words, people develop trust, maybe gradually, in certain contexts. Adler confirmed that the context in which trust is affected is part of the assessment of the trustee [17]. With respect to regulation, context can both have subtle or powerful effects on the inspectee’s action, and therefore on the inspector’s assessment. Clearly, whatever trust causes partly depends upon its context.

Meurs found that the most **important elements** for **trust** are **competencies** and intentions together with a third element, past performances, which **are the result of** the final actions that follow competencies and intentions [24].

Some researchers argued that integrity is the condition most similar, and important, to trust. The concept is depicted frequently in literature on integrity as a moral one [30]. This means that a moral commitment is deemed as a prerequisite for having integrity, hence a self-imposed binding commitment to moral values and principles which guide the inspectee’s actions.

We approach trust from the inspector in the inspectee, by using Rousseau’s definition above. This definition is dominant in organizational research and since regulatory relations are inter-organizational relationships, this seemed appropriate. We also use the different elements that came out of the studies above as a starting point (Table 1).

## Methods

The research was conducted between January 2016 and March 2017 by using a qualitative approach that combined document review, expert interviews, inspector interviews, and observations in the work meetings of inspectors within the Inspectorate.

We assembled policy documents to unravel how the issue of trust regarding regulation in Dutch care services evolved. We approached four external experts drawn from our document analysis in order to access their specific knowledge. These experts work in the fields of behavorial sciences, public health advisory boards, public governance, and regulatory supervision of education. Consulting the empirical knowledge of these experts in understanding trust in organisations in general complemented our theoretical background. In addition, we selected inspectors from the different domains of the Inspectorate, including social care, curative care, youth care, and medicinal products and equipment. We also selected inspectors for semi-structured interviews with diverse characteristics, such as gender, age, and experience in regulation.

The respondents (*n* = 11 inspectors) provided narratives from various angles. We interviewed inspectors until we reached a saturation point. The interviews were recorded with the permission of the respondents and were transcribed verbatim. Furthermore, we collected additional data from observing four regular work meetings of inspectors. During these non-participant observations, the observer had limited interaction with the inspectors she observed. The observer examined, with respect to trust in the inspectee, the details of how inspectors talk and behave together.

A priori codes were developed from the extraction of the integrative literature review for the assessment of trust detailed in the theoretical framing (Table 2, left column). The data collected from interviews and observations were analyzed using both an inductive development of a priori codes (Table 2, left column) as well as a deductive coding scheme (Table 2, middle column). We used the data from both the interviews and the observations to revise Rousseau’s definition of trust into one that matches the inspector’s experiences, and also to develop a conceptual model with all the key elements of trust for the Inspectorate (Table 2, right-handed column). In addition, we worked out a Framework of Trust for the Inspectorate, which includes practical examples associated to the key elements (See Additional file 2 for the interview format). This framework was tested and revised using four, randomly chosen, inspectors during a second round of interviews. We tested whether the framework met these four inspectors’ needs. In addition, we discussed the framework by interviewing three care providers who worked in three different types of care services, handicapped, elderly, and hospital services. These care services were chosen by the individual’s role (professional and management board member), their level of experience (senior) and, diversity (gender, professional background). The main question was whether they experienced this framework as a logical and just instrument. It allowed these care providers to play an active role in the interpretation and presentation of the Framework of Trust, and to have face to face discussions with them. The framework was also tested by using examples of specific cases in workshops with inspectors regulating different types of care services such as a hospitals, care homes, or dental services.

**Table 2:** The a priori coding scheme used for analysis in the research study. This is shown in the left-handed column. The a priori codes were developed from the extraction of the integrative literature review for the assessment of trust detailed in the theoretical framing. The middle column shows the final codes after analyzing the interviews with experts and inspectors. The right-handed column indicates the key elements after analyzing the observations and a second round of interviews

A priori codes (start list from literature)	Final codes (after analyzing interviews)	Key elements (after analyzing observations and second round of interviews)
Integrity	Integrity	Integrity
Honesty		
Caring		
Harmful		
Relation		
Adequately treated		
Openness	Openness	Transparency
Transparency	Transparency	
Accountability	Open communication	
Reflexivity	Ability to learn	Ability to learn
	Willingness to learn	Accepting feedback
Behavior	Behavior	showing actual changing in behavior, following the feedback
Intentions	Intentions	
Competencies	Competencies	
Expectation	Expectation	
Responsibility	Accountability	
Performance	Open communication	
Actions	Control	
	Improvement capacity	
Circumstances	Prediction	context
Certainty	context	
Dependency	past performances	
Control		
Risk taking act		
Experiences		
Past performances		
Vulnerability		
Reason		
Routine		
Perception		

## Results

We identified a set of potential elements (coding scheme) by using both the literature search and empirical research. From these, we revised the initial elements into a list of key elements (Table 2, right-handed column). Some existing elements merged, for example, competencies and intentions became behavior, and openness and transparency became a joint element transparency. Other themes needed to be condensed into smaller units, for example, accountability condensed into transparency, the ability to learn, and accepting feedback. Overall, the analysis revealed that we could reduce the initial elements into five which, in turn, we viewed as behavioral elements: [1] showing integrity [2]; transparency [3]; showing the ability to learn [4]; willingness to accept feedback, and [5]; showing actual changing in behavior, following the feedback. The sixth element concerned context information.

### Key elements of trust

In line with the literature, inspectors highlighted that it was problematic to define, conceptualize and operationalize the concept of trust. Personal and organizational trust were closely intertwined, as persons, such as care providers and managers, and organizations, such as care homes and pharmaceutical companies, may both be objects of trust. Trust was based on both organizational and personal trust, of which trust in the organization often referred to the people on the management board who were responsible for the organization. Furthermore, it was evident that the term trust was used inconsistently, and boundaries between elements were blurred.

Some of the inspectors used trust as the foundation of their regulation.

***‘****Trust is the foundation of my work. It is an essential part of who I am. This is necessary, otherwise you act from a place of distrust. Then you don’t do justice to the facts. So, if I visit a healthcare organization or if I have an interview with a care provider, I act from a place of trust.’*

However, not all inspectors share this idea:

*‘Beginning with assuming trust from the start is, for me, a bit naïve. In daily practice it doesn’t seem to work that way, that you can begin each interaction from a place of trust.‘.*

The inspectors statements contradict each other in what their attitude towards the inspectee should be. Some argue that trust is positive and distrust is destructive, while others argue that distrust is rational and trust naïve. The majority of the inspectors share the idea of responsiveness, however it depends on context and their field of work.

1. *Showing Integrity*
  *‘I noticed that the board member has been guided by his own interests and not by the interests of the clients for which he functions’.*

Another important element, for inspectors, was integrity. Here the inspectors referred to a caregiver’s need to be committed to moral principles and values that satisfy a minimum standard of care from a moral perspective, or ‘standing for good care ’. This is closely related to the demand for honesty which indicates that a person’s commitments and actions should reflect who they are and what they stand for. And also to the elements of transparency, accepting feedback, and showing actual change in behavior presented below. Where there is some question of a lack of integrity, the inspectors we interviewed also associated this with fraud, corruption, a conflict of interest, and inconsistency in behavior. Therefore, integrity played an important role in the field of healthcare fraud.

*‘My sense of trust in the care provider is diminished as soon as I have facts about healthcare fraud’.*

Inspectors know that if healthcare fraud is committed, the abusive practices lead to poor and inadequate care and vulnerable patients and clients. One inspector stated that he himself tried to trust the inspectee as much as possible, but working in the field of healthcare fraud meant it was often difficult to avoid prejudice and to shun any initial mistrust. We also noted that this concept is driven by consistency in the inspectee’s behavior. Sometimes, inspectees say they want change and continual improvement but they do not follow through. For example, when the Inspectorate identifies inconsistencies during a review of medical reports [31]. Consistency is essential for trust and trust is essential for any healthy relationship [32].

2. *Transparency*

*‘I found it encouraging to hear from the client council that the board member had an open dialogue with them and informed them in time’.*

Here the inspectee showed the ability to engage in an open and transparent conversation with one of their stakeholders in a meeting. The skill of being open builds on the skill of being transparent. Transparency is especially important when serious adverse events occur as the risk of the care organization not responding to such events will cause a loss of trust. Whilst inspectors were keen to stress the importance of openness and transparency, it was acknowledged that this element was a difficult concept to grasp and to distinguish from accountability. Both concepts go hand in hand with open communication.

3. *Showing the ability to learn*

*‘ I feel trust in this health-care organization because they investigated the adverse event very carefully, they consulted the literature, and relevant actions were taken’.*

If the inspectee shows the ability to learn and the ability to improve the quality of care, this had an impact on the inspector’s level of trust. This is in line with the responsive regulation theory which is more suited to organizations and sectors seeking long-term improvement, rather than short-term compliance.

4. *Showing willingness to accept feedback*

*‘I feel a trust in his ability to reflect on his own actions if I see that it brings new insights’.*

The Inspectorate can give feedback on the actions, and the results provided by the inspectee. The Inspectorate assesses whether the inspectee is able to reflect on their actions. By providing time for reflection and evaluations, the care provider showed that they accepted feedback and took it seriously. Inspectors found it essential that healthcare services show a culture of regular reflection and asked for feedback, for example by information solicited in a **360-degree feedback** process from an employee’s peers (colleagues) and supervisors.

This behavior follows a variant of responsive regulation, namely smart, or self-regulation, which suggests that this regulation encourages self-reflection about performance and therefore an ongoing improvement of the quality of care.

5. Showing actual changing in behavior, following the feedback.

*‘I‘II appreciate it when the care provider wants, and is able, to explain why he didn’t comply with the expectations and regulations’.*

Inspectors also stressed that the care provider should explain their actions to external and internal parties such as the Inspectorate, works council, client council, employees, and colleagues. To clarify why an instance of unsafe care had occurred, the inspector should discuss the matter with the inspectee and ask questions to clarify the facts and circumstances. This suggests that regulatory agencies developing responsive models of regulation need to develop their assessments to understand the facts and circumstances, and to develop regulatory responses, including information sharing and continuous reflection, to assess performance.

### Context

Inspectors explained that information about context can have a positive or negative influence on their level of trust in the inspectee. Inspectors brought examples of the organizational, that is internal context which had a negative effect such as high staff absences due to sickness or a change in the management board. An example of organizational context which had a positive effect on the level of trust was a healthcare organization which had organized and facilitated a process of effective reflection for their employees. Another example was an increasing regional demand for healthcare, for example due to an ageing population, which was explained by information on the social context. This demand for healthcare affected the expectation that the care provider was able to deliver good care and had an impact on the level of trust in the care provider.

### Definition and conceptual model

During the interviews, we discussed the definitions and descriptions of trust proposed by the authors presented in Table 1. These discussions and consequent readjustments led to the following definition of trust from the Inspectorate’s perspective: *Trust is the expectation by the Inspectorate that the behavior of the inspectee demonstrates a commitment to good care and shows how they can contribute to this commitment in their own context.*

**Fig. 1:**
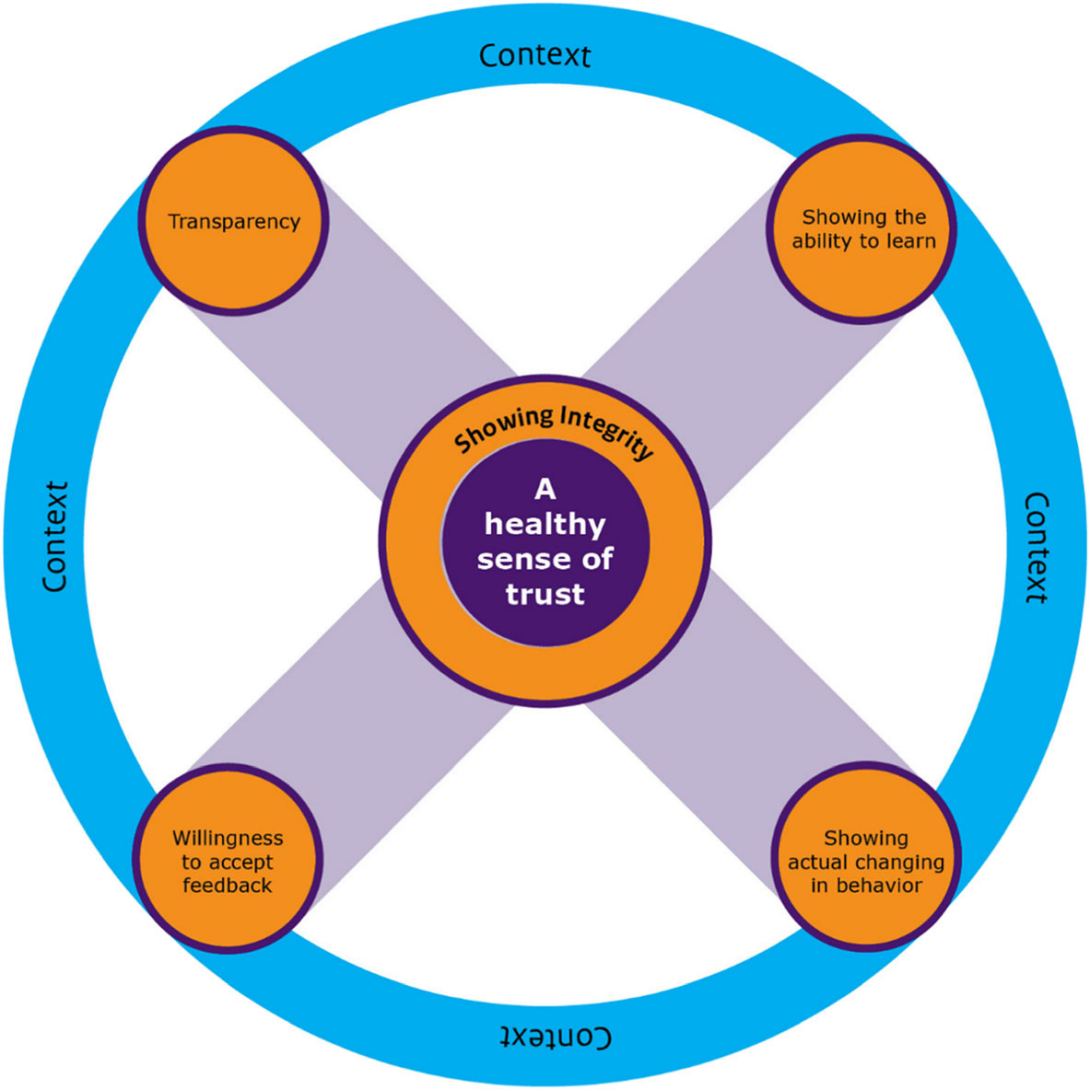
Conceptual Model for Trust from the inspector in the inspectee

The six key elements, which build on the literature search and the empirical data, were merged into a conceptual model (Fig. 1). In the center of the model is a sense of trust. It is in the center because that is the foundation of the Inspectorate. The orange circles are the five key behavioral elements. The two pairs of elements are connected in the model. The first pair is ‘showing the willingness to accept feedback’ and ‘showing the ability to learn’, are behaviors which match self-reflection; the second pair is ‘showing actual change in behavior (following the feedback)’ and ‘transparency’, which match open and corrective actions. The element integrity has a central place in the model because it is strongly interrelated with the four other behavioral elements [33]. The blue outer circle symbolizes the context of an inspectee’s situation.

### The framework of trust

The Framework of Trust builds on the literature search and the empirical data presented [34]. It includes practical examples associated with the key elements presented by the inspectors whom we interviewed. For example, with respect to integrity: ‘Does the inspectee avoid inappropriate relationships and conflicts of interest?’ These examples enable other inspectors who use this framework to express themselves more explicitly and consistently with respect to trust. It has been presented as an interactive PDF document to aid navigation through the framework. Users of the framework can click on one of the key elements in the model in order to navigate to other relevant information, such as a definition or a description from the literature. Practical examples of this element drawn from the inspectors interviewed are also available. Table 3 shows the definitions of the five behavioral elements based on literature. Care providers of services who were interviewed to give feedback experienced the Framework of Trust as logical and justified, confirming our findings.

**Table 3:** Definitions of the five key elements with respect to behavior in the model

Behavior	Definition
Showing integrity	The inspectee acts in the interest of the organization and/ or society in accordance with generally accepted standards and values [15, 33, 35].
Transparency	The inspectee has an open attitude and shows a willingness to share information and communicate honestly both internally and externally [36, 37].
Ability to learn	The Inspectee shows an ability to learn from experience and feedback and applies this changes to new situations. With this in mind the inspectee takes deliberate steps to learn and improve performance by mobilizing resources and implementing improvements [38, 39].
Willingness to accept feedback	The inspectee shows a willingness to accept criticism from internal and external supervisors and to comply with relevant recommendations [40].
Actual changing in behavior, following the feedback	The inspectee shows responsibility for his/her actions and clarifies their behaviors and takes necessary action to rectify [41, 42].

## Discussion

We investigated how the inspectors interpret and use the concept trust in the inspectee. Six elements with respect to trust emerged from the data: [1] showing integrity [2]; transparency [3]; showing the ability to learn [4]; willingness to accept feedback, and [5]; showing actual changing in behavior, following the feedback. The sixth element concerned context information. These five behavioral elements, plus the context information were merged into a Framework of Trust and designed into an interactive PDF document.

### The relationship between the empirical research and literature

Of all elements of trust identified in our empirical research, the inspectee’s ability to learn and improve dominated in practice. This, together with accepting feedback, represented an emphasis on reflectiveness. Both refer to recognizing the need to develop the ability to learn and reflect upon improvement. A possible explanation for the dominance may be that over recent years the Inspectorate has moved from compliance to an approach more focused upon responsiveness. This idea matches the growing emphasis by the Inspectorate on organizational learning and the safety culture of the inspectee [10].

Our empirical research reflected that from the literature study in concluding that behavior is an important element of trust. According to Rousseau, the development of trust is based upon the positive expectations of the intentions [15]. Inspectors acknowledged that it starts with intentions because signs of positive intentions are necessary for the trusting party to be able to accept a potentially vulnerable position - one that is risk inherent [15]. Positive intentions appear through signs of cooperation and a partner’s active behavior. However, inspectors emphasized that intentions are difficult to verify while actual behavior can be observed. Behavior is the sum of the visible and invisible actions of a person, whereas performance is the result of all actions. From a regulatory perspective the final results are of most significance.

### Reflections on results

The responsive model assumes that the Inspectorate has the interpersonal and reflexive competencies to choose appropriate enforcement strategies and thus to communicate clearly with inspectees [43]. This means that inspectors should share the level of trust they have in the inspectees explicitly and logically. Reflexive competencies are also needed if building and repairing trust is to be successful, both for the inspector and the inspectee. The majority of inspectors use trust as a foundation in their supervision. Inspectors with distrust of inspectees, however, will most likely signal their distrust to inspectees, probably without being aware of it. As Mascini and van Wijk showed: “A negative relational signal frequently dominated an entrepreneur’s overall perception of the inspector’s conduct and resulted precisely in the negative effects the inspectors were trying to prevent with their predominantly persuasive approach” [44].

Biases influence the way in which people make assessments and, therefore, influence their actions [45]. Biases arise from starting and then suspending thoughts and evaluations. In situations of trust, it is reasonable to assume that these are also put into practice as a result of the trustor making sense of their situation of trust [46]. It is important that inspectors are aware, both of their own biases, and those of their colleagues, and make their trust in the inspectee explicit. By using the Framework of Trust it is possible to minimize the impact of biases on the effectiveness of regulation.

## Conclusions

This research study focuses on the regulation of care services. It explores from inspectors’ perspective how trust in the inspectee is conceptualized and assessed. We proposed a practical framework of trust in order to reduce the conceptual ambiguity, strengthen regulatory assessment, and support appropriate tailoring of the regulatory response. This study reveals that there are multiple ways in which the concept of trust can be defined. We synthesized the concept of trust into six elements containing five types of behavior and information about their context. Empirically, this research study has, we believe, addressed a gap in the knowledge regarding the assessment of trust in the inspectee. Other Inspectorates may learn from these results for their own practice and may explore whether operational deployment of the Framework of Trust effects their assessment and enforcement strategies. We, therefore, call for further pragmatic and reflexive experimentation and research into the Framework of Trust in regulation by other regulatory agencies.

## Supplementary information

**Additional file 1.** About the Dutch Health and Youth care Inspectorate

**Additional file 2.** Interview format for the semi-structured interview

## Data Availability

Participants gave no consent for data sharing.
