# Supplementary material for: An empirical study of how the Dutch healthcare regulator first formulates the concept of trust and then puts it into practice
Source: BMC Health Serv Res. 2019 Dec 10;19:951. doi: 10.1186/s12913-019-4797-3 (PMC6905045; doi:10.1186/s12913-019-4797-3)
Supplement: Supplementary file 1 — Additional file 1. About the Dutch Health and Youth care Inspectorate [file 12913_2019_4797_MOESM1_ESM.docx]

***Appendix 1:* About the Dutch Health and Youth care Inspectorate**

| The Dutch Health and Youth care Inspectorate (hereafter the Inspectorate) is part of the Ministry of Health, welfare and Sport of the Netherlands. It is the official regulatory body charged with supervising the quality and safety of healthcare services, prevention activities and medical products. The Inspectorate has organized its regulation in several ways in order to ensure compliance with professional standards and guidelines, and to ensure patient safety.  The most important methods are supervision based around incidents and analyses of various types of risk information, also known as risk-based regulation. If risks are identified, then the inspectorate visits a care provider. This method of supervision consists of general supervision and, what is defined as thematic supervision. In this case the Inspectorate conducts visits to several organizations based on a specific theme common to the whole sector. This action is the result of research or incidents such as medication safety. The Inspectorate then assesses one or more risks depending on the diversity and severity of the risks identified within a specific organization. This assessment is based on reviewing documents relating to the quality management systems, such as care protocols and patient files, but also to reviewing the communications with, for example, care managers, care professionals and patients. The organization must then take measures to improve safety and thus reduce the risks. These improvements and their results must then be reported to the Inspectorate. The Inspectorate will then conduct a follow-up visit to assess the implementation of the improvement measures. The inspectorate is mandated to use enforcement measures if the organization does not comply and there is insufficient faith in the organization to realize the improvements in time. For instance, the Inspectorate can impose intensified supervision of an organization. This may involve frequent visits, announced or unannounced, and consultations with the board. They can involve the organization as a whole or just one of its departments. The inspectorate can also penalize the organization, including prohibiting it, temporarily or even permanently, from accepting new patients. |
| --- |
