# Supplementary material for: An empirical study of how the Dutch healthcare regulator first formulates the concept of trust and then puts it into practice
Source: BMC Health Serv Res. 2019 Dec 10;19:951. doi: 10.1186/s12913-019-4797-3 (PMC6905045; doi:10.1186/s12913-019-4797-3)
Supplement: Supplementary file 2 — Additional file 2. Interview format for the semi-structured interview [file 12913_2019_4797_MOESM2_ESM.docx]

Appendix 2: Interview format for the semi-structured interview

| **Main questions** | **Clarifying questions** |
| --- | --- |
| In general, what do you think of trust as a foundation in regulation? | Can you please expand a little on what you said? |
| How do you interpret and use the concept of trust in the inspectee in your daily practice | What makes it difficult/not difficult for you to use this concept of trust? |
| What do you think of this definition? ‘*Trust is a psychological state comprising the intention to accept vulnerability based upon the positive expectations of the intentions or behavior of another’* | Does this definition meet your definition? If so or if not-> why?  Which different elements are essential or characteristic parts of your definition?  Which elements are missing according to your definition? |
| In particular, what do you think of the codes in the a priori coding table? | Can you please expand a little on each code?  Can you please give some practical examples of this code?  Which different codes are essential or characteristic parts of your daily practice?  Which elements are missing?  Which codes are superfluous? |
|  |  |
